# Versatility of composite grafts for nasal defects – a case series

**DOI:** 10.1080/23320885.2022.2140665

**Published:** 2022-12-01

**Authors:** Caroline Asirvatham Gjorup, Michael Prangsgaard Moeller, Camilla Asklund, Lisbet Rosenkrantz Hölmich, Line Bro Breiting

**Affiliations:** Department of Plastic Surgery, Copenhagen University Hospital, Herlev, Denmark

**Keywords:** Alar reconstruction, composite graft, chondrocutaneous graft, facial reconstruction, nasal reconstruction

## Abstract

This retrospective study describes the reconstruction of 18 nasal defects with chondrocutaneous (composite) grafts. Composite grafts are versatile one-stage options for defects ≤2.5 cm at the lower third of the nose and are particularly useful in reconstruction of small full-thickness defects and superficial defects bordering or involving the alar rim.

## Introduction

Reconstruction of nasal defects involving the nasal alar or soft triangle can be challenging due to the complex three-dimensional anatomy of the nose with high functional and esthetical demands. Soft-tissue reconstruction without a structural component can lead to collapse of the nostril, causing stenosis. This lack of structural support can also cause a cephalad retraction of the alar rim resulting in an aesthetically unpleasing outcome, and uncomfortable exposure of nasal mucosa. These challenges can be managed with a composite graft, consisting of cartilage, subcutaneous fat, and the overlying skin, providing stability from the cartilage and the skin needed to cover the defect, for both superficial and full-thickness nasal defects. Using a composite graft from the ear provides tissue with a 3 D-structure, color, texture, and thickness matching the nose to manage both the aesthetic and functional demands. This cartilage provides stability to not only avoid alar retraction but also ensure a patent airway. The auricular donor site allows for the graft to be harvested with skin on both sides if inner lining is needed for full thickness defects. Furthermore, it provides a well-tolerated donor site away from the midface.

Composite grafts were first described more than a century ago [1]. Their use is versatile and can be used for primary and secondary reconstructions, and as salvage procedures to correct functional and aesthetical impairments. The ideal indication for a nasal reconstruction using a composite graft harvested from the ear is for alar or soft triangle defects up to approx. 1–2 cm involving or close to the alar rim with superficial or full-thickness defects. The drawback of the composite graft is its dependence on revascularization which limits the size of full thickness defect possible to reconstruct with a composite graft, as well as the potential shrinkage or atrophy.

At our unit, the use of composite grafts has increased since 2017 and this case series will present the lessons we have learnt. We describe our series of patients with nasal defects bordering or involving the alar rim managed either primary after surgical resection or secondary to correct alar retraction or stenosis with composite grafts harvested from the auricle.

## Methods

This study is a retrospective study of eighteen consecutive patients who had nasal reconstruction performed with composite grafts at the Department of Plastic Surgery at Herlev and Gentofte Hospital, Copenhagen University Denmark, from July 2017 to February 2022. Data were extracted from the electronical recorded medical notes (Epic Systems Corporation) and clinical photographs. This study was undertaken as a quality control, with the patients’ written informed content, and approved by the hospital administration and no ethical approvement was therefore necessary.

### Surgical technique

**Figure 1.**
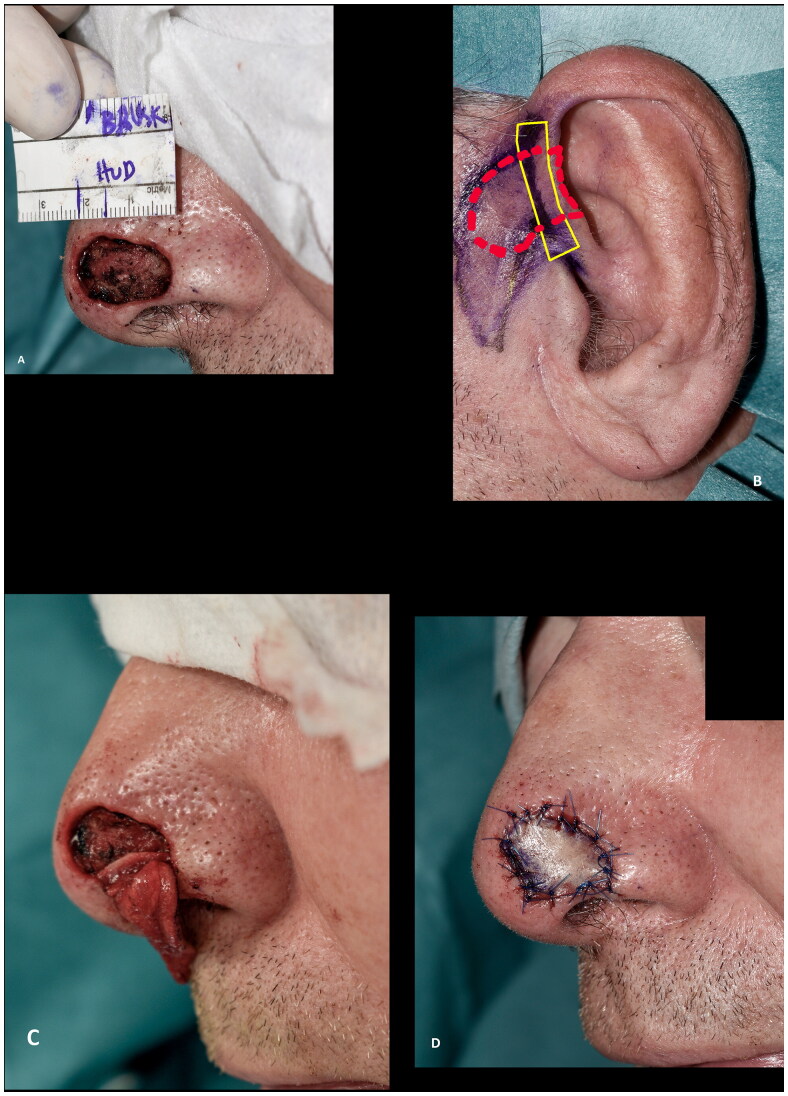
Surgical technique. (A) Recipient site after radical resection of basal cell carcinoma. (B) Preoperative marking at the donor site. Note the marking of the harvested cartilage (solid line) which were approximately 3–5 mm in height and 3–5 mm in length and the skin (dashed line). (C) Cartilage wings in place, secured in subcutaneous pockets parallel to the alar rim with dissolvable sutures. (D) Composite graft in place.

The surgical procedure was generally performed as described: The recipient site was cleared of cancer or prepared for the corrective procedure. Careful hemostasis was achieved, limiting the use of cautery to avoid closing the dermal and subcutaneous vessels necessary for graft take. The cartilage graft was then marked on the donor site with the width of the defect at the alar rim 1:1 adding another approx. three to five mm of cartilage at each end as shown with the solid yellow lines in Figure 1(B). These stabilizing limbs (also known as wings, pegs and struts) of cartilage without overlying skin is used for securing the graft at the recipient site. The skin is hereafter marked overlying the cartilage as shown with the dashed red lines in Figure 1(B). For practical reasons, the ipsilateral ear was mostly used if the shape of the cartilage was satisfactory. If two surgeons were operating, or if the patient wore a hearing aid on the ipsilateral side, the contralateral ear was chosen. The root of helix or the posterior aspect of the concha were used. The purpose of the limbs was to avoid a notch at the edge of the alar rim of the defect, to increase the surface area of revascularization, and to increase graft stability, with decreased shearing forces of the graft over its recipient bed [2]. The skin markings can be elongated to include non-hair bearing preauricular skin if needed (Figure 1(B)). The skin overlying the cartilage wings was incised but not excised to allow for direct closure over the donor site of the limbs, minimizing donor site morbidity. Utmost care was taken to avoid separation of the loosely adhered skin and cartilage. In some cases, this was done by securing the skin to the cartilage with a 6–0 or 7–0 temporary suture placed during harvesting of the graft. The stabilizing limbs were approximately 3–5 mm in height and 3–5 mm in length. Once the graft had been harvested, the donor site was closed directly or by mobilising preauricular tissue as an advancement or rotational flap or by advancing the helical rim as an Antia-Buch flap [3]. At the recipient site, subcutaneous pockets were prepared along the alar rim and the stabilizing limbs of the cartilage were inserted into the recipient site as an ‘interlocking graft’ [2]. If needed, the stabilizing limbs were fixed to the skin and/or wound bed with sutures. The skin graft was fixed in a standard fashion, typically with 6–0 or 5–0 Nylon sutures. If the defect was not full thickness, the graft was fixed with a bolster dressing. If the defect was full thickness, a nasal airway, or an inner nasal compression was fixed to the nose and/or upper lip to eliminate dead space, immobilize the graft, and to preserve the shape of the nasal ala during healing.

## Postoperative care

Postoperative restrictions were advised until the dressing was removed (after 7 days) to avoid haematoma. If there was any concern about graft take, or epidermolysis, the patient was seen regularly until the graft had taken or re-epithelialized. Depending on the preferences of the surgeon, prophylactic antibiotics were prescribed. Some surgeons advocated for postoperative cooling of the graft (such as 10–15 min/hour for the first 24 h). All patients were followed closely, and if the patient and surgeon were satisfied with the outcome at 3 months follow-up, most were transferred for follow-up at their general practitioner or dermatologist.

### Statistical methods

All results are descriptive and shown as number (%) and mean (range).

## Results

**Figure 2.**
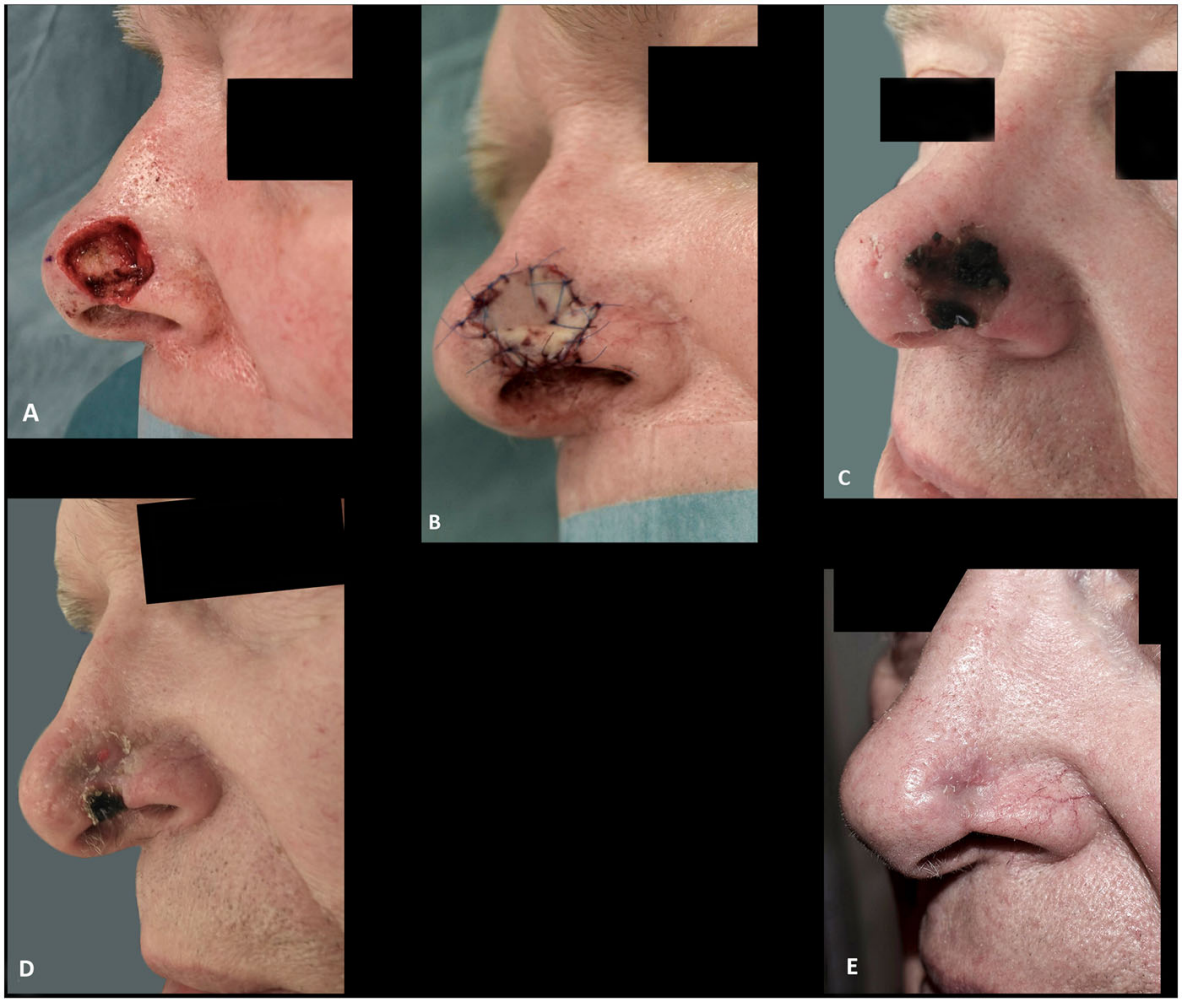
(A,B) A defect after radical resection of basal cell carcinoma involving the alar rim reconstructed with a composite graft. (C) Epidermolysis eleven days after surgery. (D) The graft at two months follow up. (E) Despite the partial graft loss and alar retraction at three months follow up the outcome was rated as acceptable.

**Figure 3.**
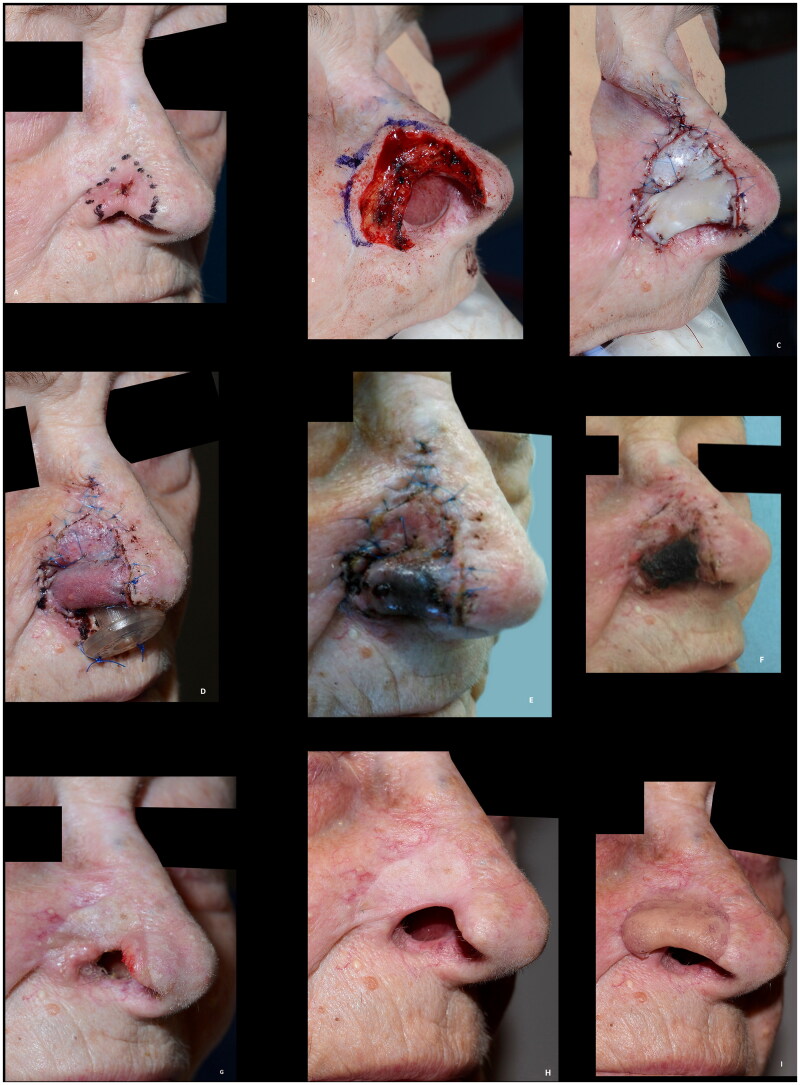
(A) 81-years-old female with a basal cell carcinoma causing alar retraction prior to surgery. (B) The large full thickness defect after radical resection of basal cell carcinoma involving the whole alar subunit and part of the lower nasal sidewall. (C) The patient declined the offered paramedian forehead flap and was subsequently reconstructed with a composite graft. A suture was placed in the center of the graft to imitate the alar sulcus, which might have added to the poor graft take, as partial graft loss occurred, despite the large wound bed, as evident on images (B–G). (H,I) The remaining healed graft, as seen eight months postoperative, provided stability for an exoprosthesis and for skin-coloured surgical tape (Micropore™) that the patient considered to be more convenient to use. The patient declined revision surgery. This outcome was rated as poor.

**Figure 4.**
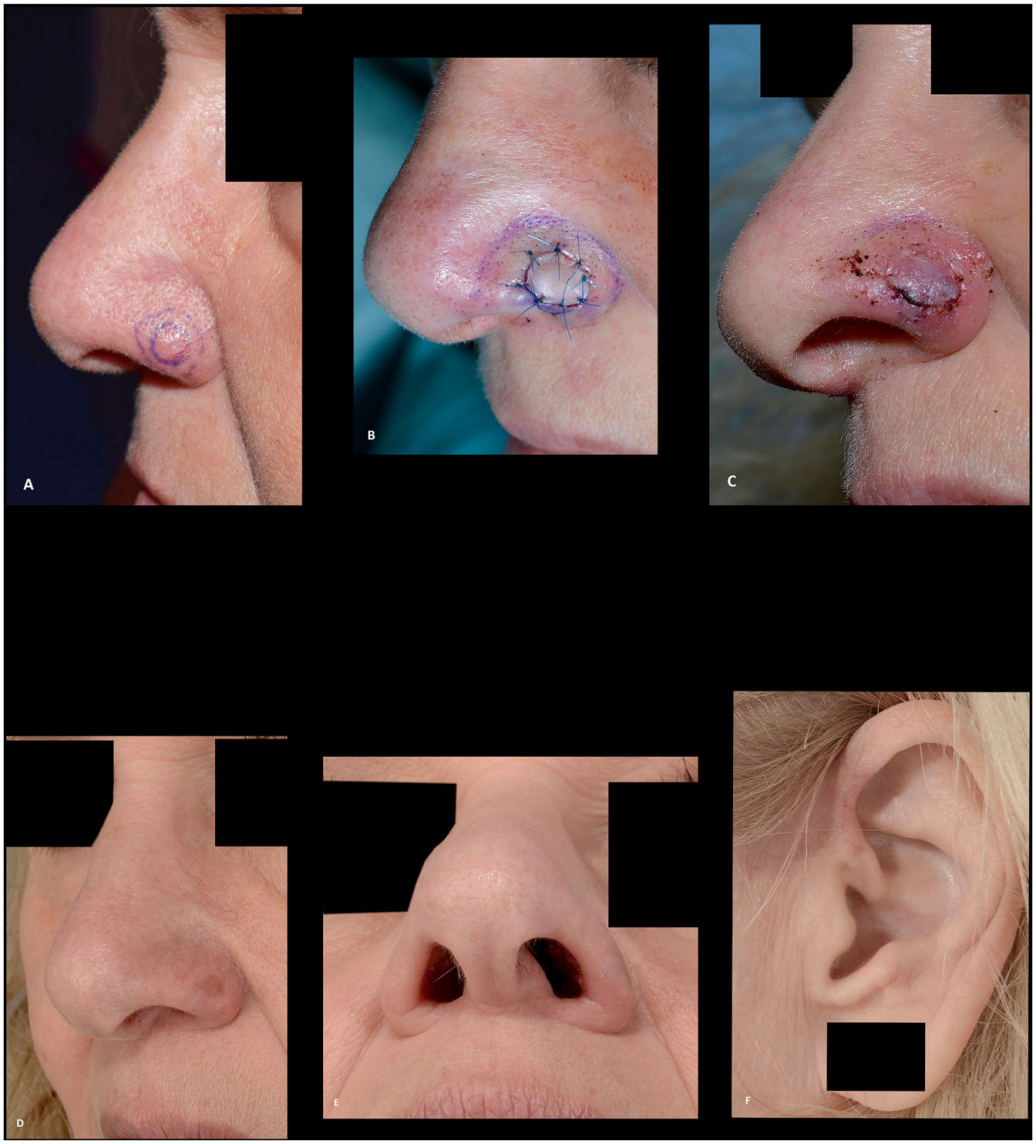
(A,B) A superficial defect, 2 mm from the alar rim, reconstructed with a composite graft to prevent alar retraction. (C) The composite graft at the first change of dressing after six days. (D–F) The results at five months follow-up, showing a good result with intact contour of the alar rim. Additionally, a good result at the donor site at the crus of helix.

**Figure 5.**
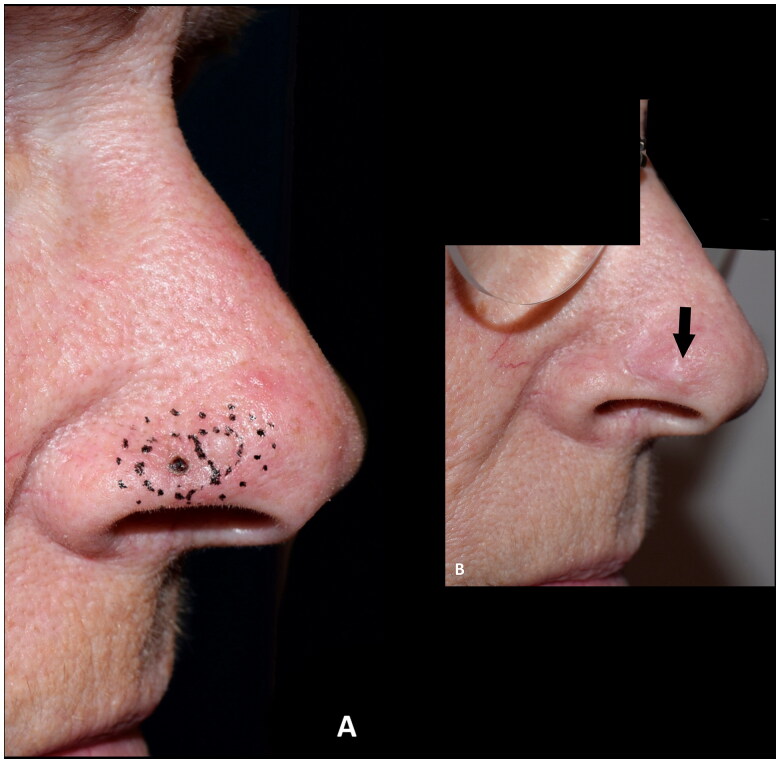
(A,B) A basal cell carcinoma, close to the alar margin, reconstructed with a composite graft maintaining the contour of the alar margin. The outcome was rated as good. This patient, however, experienced discomfort caused by the cartilage protrusion (arrow) and revision surgery was performed.

**Figure 6.**
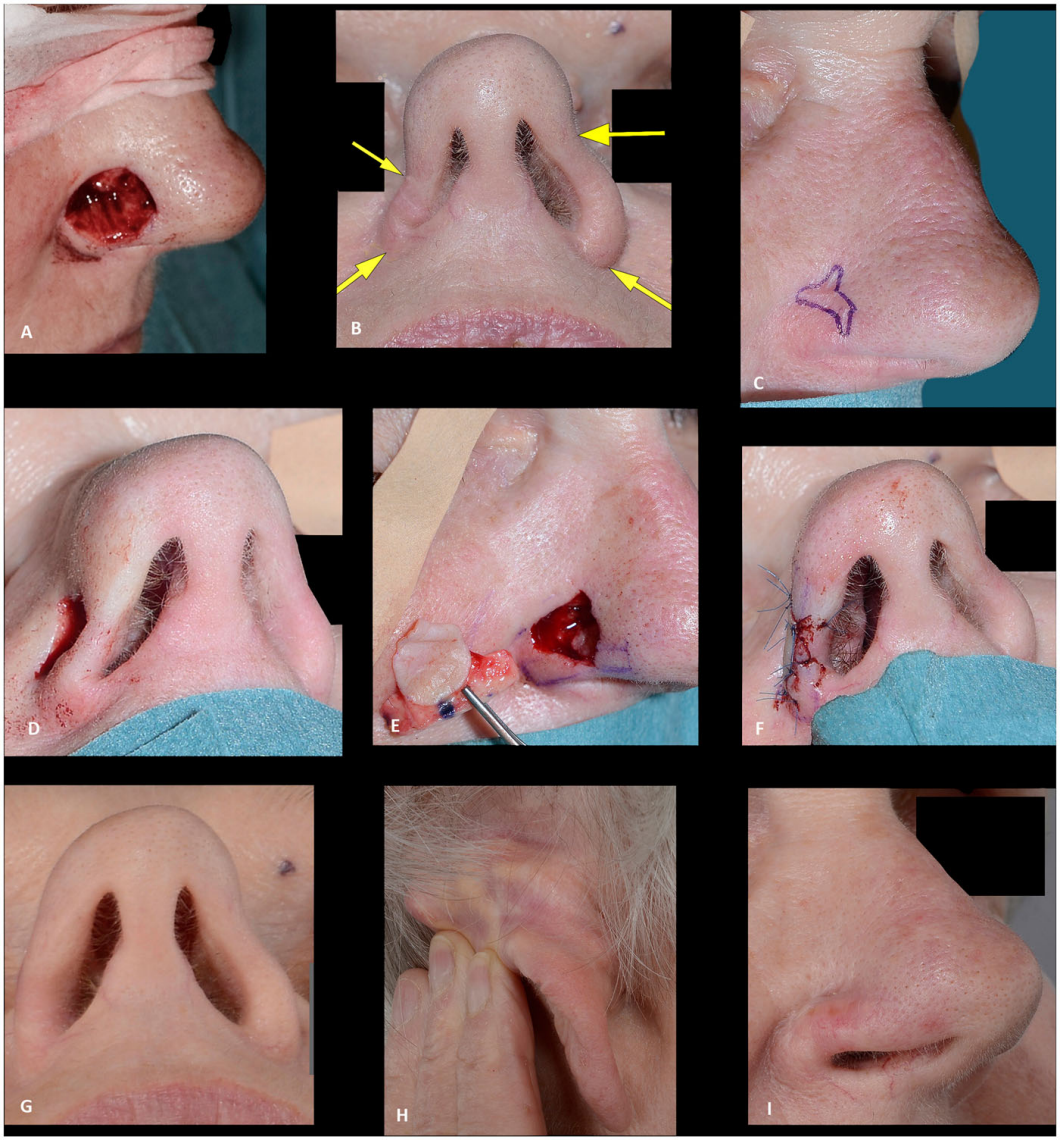
(A) Resection of basal cell carcinoma at the right alar rim, which was left to heal by secondary intention. (B) Shrinkage and collapse of the nostril led to nasal obstruction, which was evident at four months follow up. Note the shortened height of the ala in comparison to the contralateral ala (yellow arrows). (C–F) Revision surgery with a composite graft from the posterior surface of the ear was performed nine months after the defect was left to heal secondarily, successfully alleviating the nasal obstruction. Note the cautious cautery to the wound bed and the wide cartilage wings needed to stabilize the alar I. (G) An excellent functional outcome was observed three months later. (H,I) At twenty-two months follow up the functional outcome was still excellent, and the discoloration had subsided and donor site had healed well. The outcome was rated as good.

**Figure 7.**
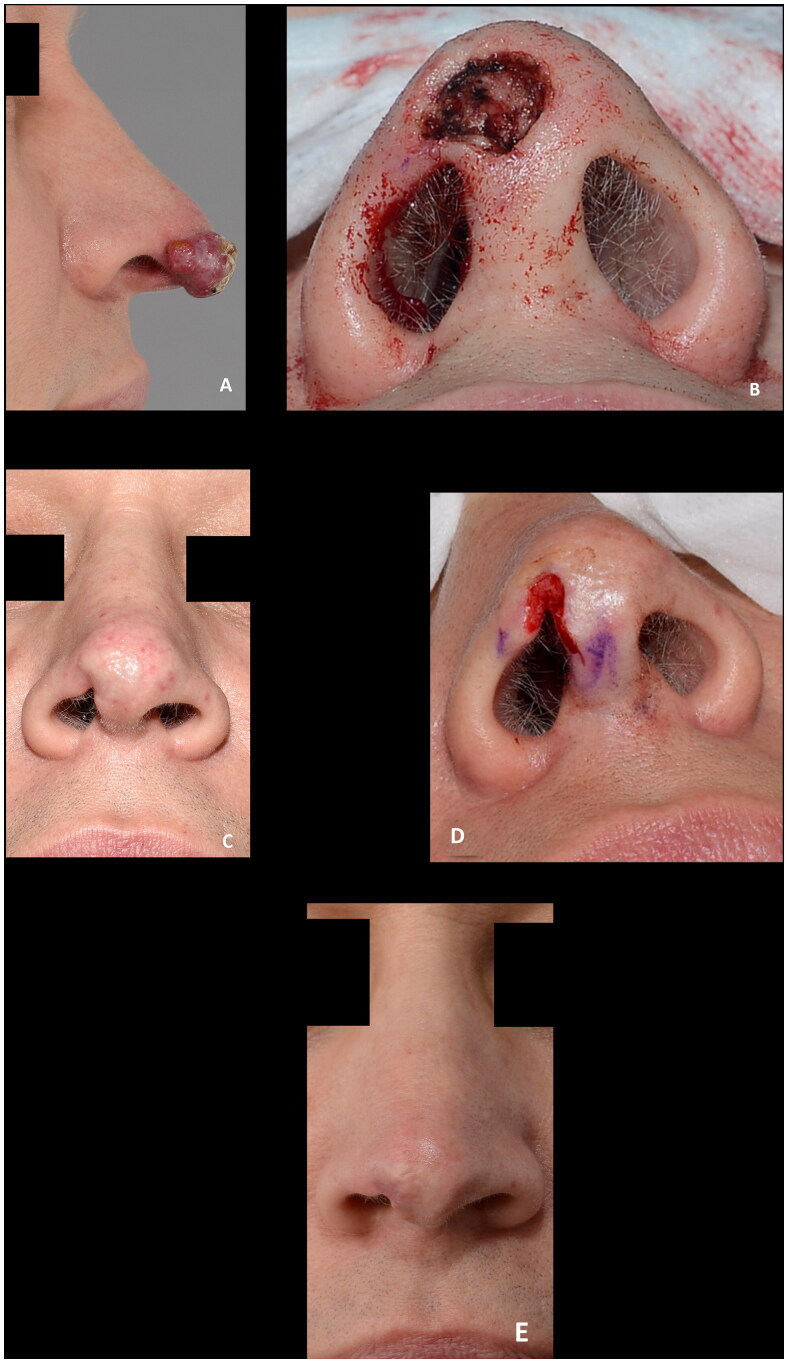
(A) A pyogenic granuloma involving the nasal tip and soft triangle. (B) The superficial defect two days later (after the final histology report was clear of the granuloma). (C) The result, three months after closure of the defect with a full-thickness skin graft, showing an unacceptable alar retraction at the soft triangle. (D–E) Nine months after the first reconstruction, revision surgery was performed with a composite graft correcting the alar retraction with a more acceptable result after three months. Note the excision of the skin above the full thickness defect, allowing for a well vascularized wound bed for graft take. The outcome was rated as acceptable due to alar retraction.

**Figure 8.**
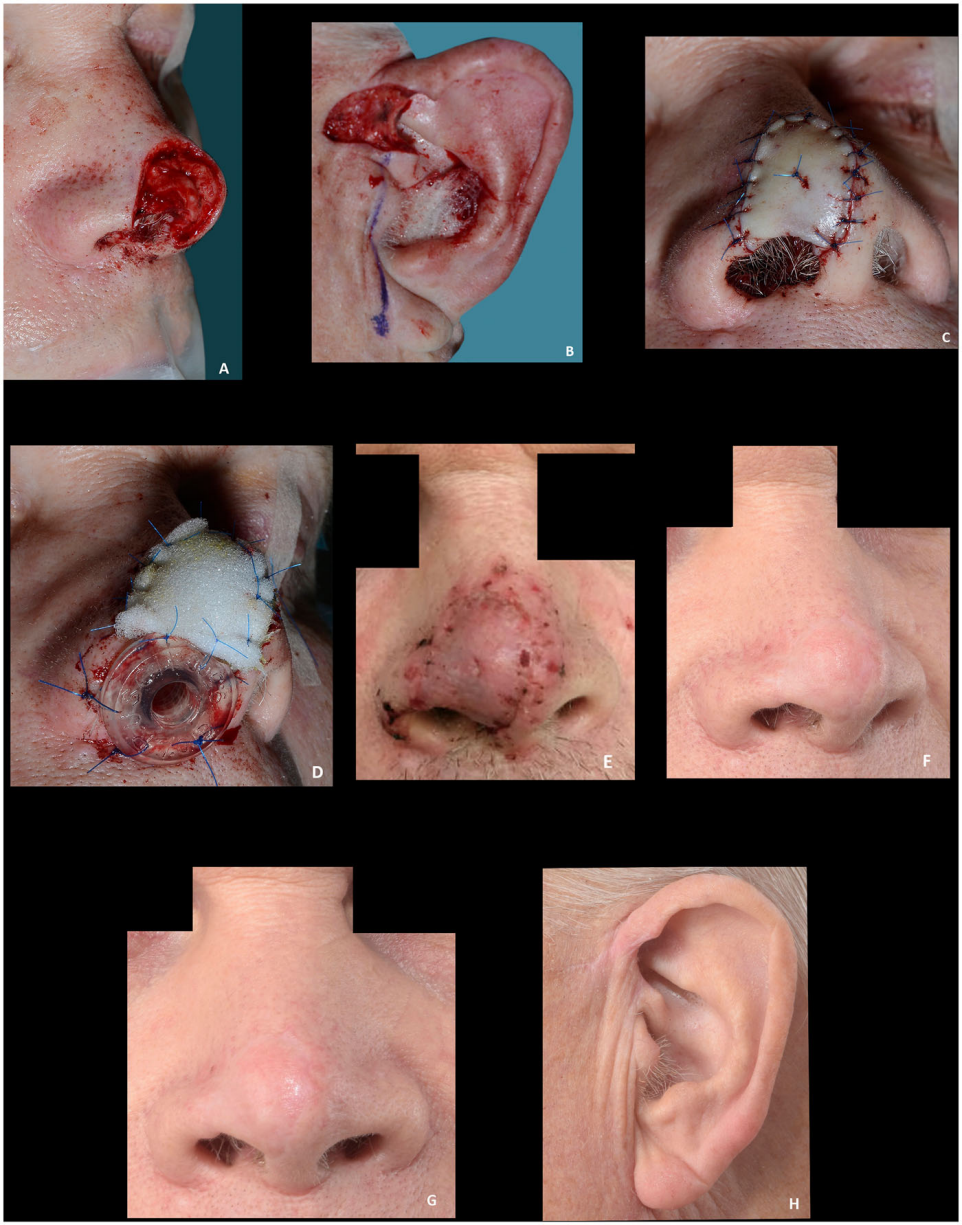
(A–D) A large superficial defect with a smaller full thickness defect measuring 4 × 12 mm, providing a well-vascularized recipient site for graft take. Note the nasal airway secured to maintain the airway, eliminate dead space, immobilize the graft and to preserve the shape of the nasal ala while the graft heals. (E) The result at the first dressing change seven days postoperative. (F–H) Fourteen months postoperative showing results rated as good. Courtesy of consultant, plastic and reconstructive surgeon Jais Oliver Berg, Dep. of Plastic Surgery, Herlev and Gentofte Hospital, Denmark.

**Figure 9.**
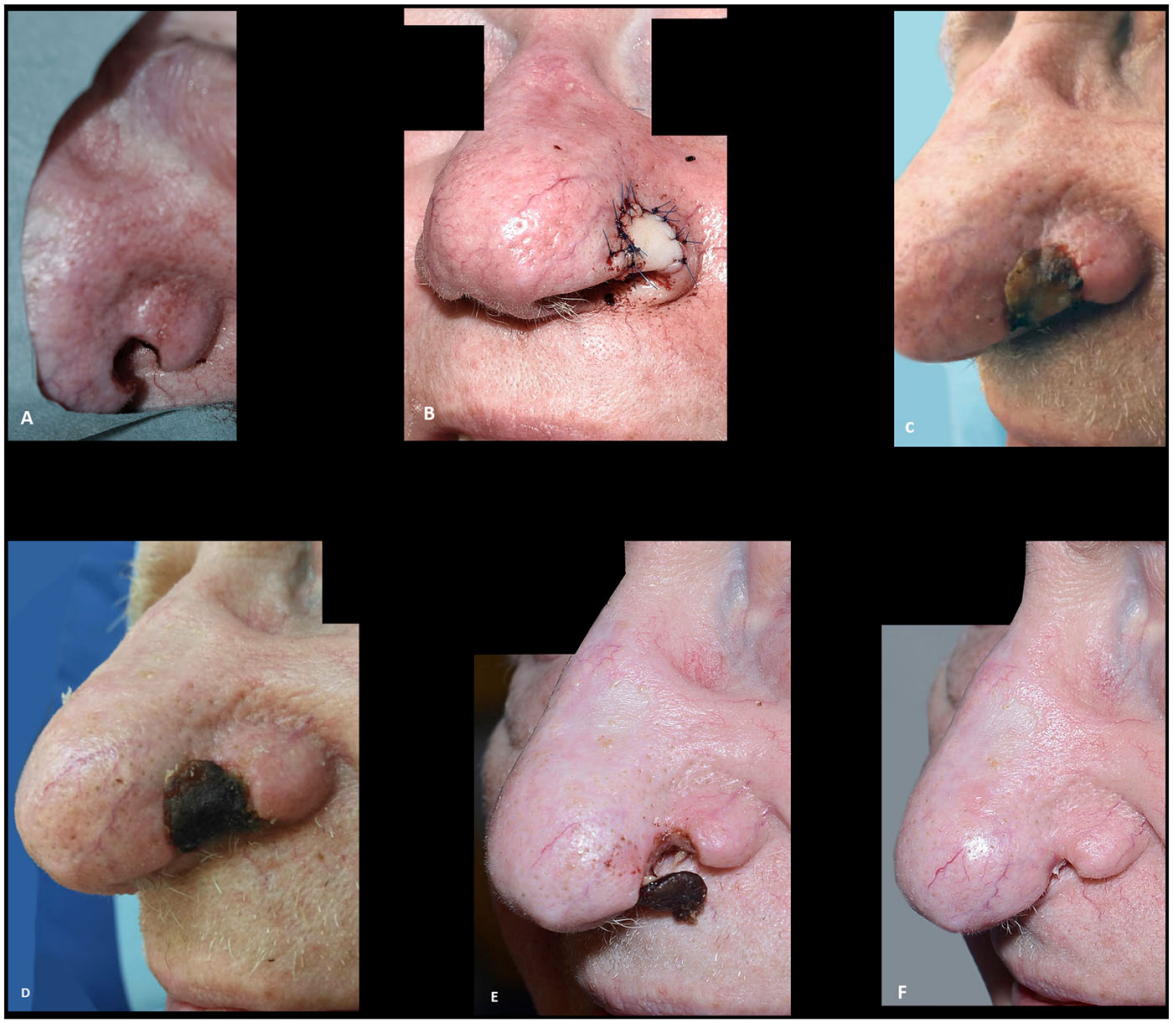
(A,B) Intraoperative view of the full thickness defect and the composite graft. (C–E) Composite graft at seven, thirteen and sixteen days postoperatively. (F) The result six weeks postoperative after secondary healing. The patient declined further reconstruction. The outcome was rated as poor.

Eighteen Caucasian patients, who had composite grafts performed by either of the authors or five additional colleagues, were included (Table 1). Most cases were performed under local anesthesia after surgical resection of basal cell carcinoma (BCC) (Figures 1–5 and 9) but grafts were also used after resection of SCC (Figure 8) and to improve functional and aesthetic outcome after previous surgery (Figures 6–7). Of the 18 patients, 12 (67%) had the procedure performed under local anesthesia. The operating time for the composite graft, after clear resection margins, was typically 1 h. The surgeons’ level of expertise ranged from registrars, reconstructing simple superficial defects near the alar rim, to senior consultants reconstructing complex full-thickness defects. The mean maximal size of the superficial defects was 13 mm (range 6–25 mm). For the eight full-thickness defects the mean width of the full-thickness defects was 11 mm (range 6–20 mm) and the mean height of the full-thickness defect was 4 mm (range 1–12 mm). The donor site was closed directly in 14 (78%) cases or with local flaps, such as minor preauricular advancement or simple rotation flaps or Antia-Buch flap in 4 (22%) cases. First dressing change was performed after an average of 7 days (range 5–10 days). The patients were on average seen three times (range 1–5) at the outpatients’ clinic by either the surgeon or a nurse until sufficient graft take was noted. No postoperative hematomas or infections occurred. Partial graft loss was seen in 3 (17%) patients, of which one underwent revision surgery with direct closure of the distal part of the defect, one healed secondary with an acceptable outcome (Figure 2) and lastly, one patient declined revision surgery and was fitted with an exoprosthesis but preferred camouflaging the defect with skin-coloured surgical tape (Micropore™) (Figure 3). Complete graft loss was experienced in one (6%) case; an 87-year-old diabetic male with a full thickness defect, where graft take was only possible from the margins of the defect (Figure 9).

**Table 1.** Demographic, procedure, and postoperative characteristics of 18 patients undergoing composite grafts for nasal reconstruction.

Patient characteristics	*n* = 18	
Age at time of surgery, mean (range)	69	53–87
Sex		
Men	8	(44%)
Women	10	(56%)
Smoking	0	(0%)
Comorbidity		
None	6	(33%)
≥1	12	(67%)
Etiolology		
BCC	15	(83%)
SCC	1	(6%)
Nasal obstruction	1	(6%)
Pyogenic granuloma	1	(6%)
Previous radiation	0	(0%)
**Proceduce**	*n* = 18	
Anasthesia	Number (%) or mean (range)	
Local	12	(67%)
General	6	(33%)
Surgeons’ level of expertise		
Registrar	2	(22%)
Fellow	3	(33%)
Consultant	4	(44%)
Number of cases per surgeon, range	2	(1–5)
**Nasal defect characteristics**	Number (%) or mean (range)	
Max dimension of the superficial defect (mm)	13	6–25
Involvement* of alar rim	10	(56%)
Full thickness defect	8	(44%)
Mean width of the full thickness defect (mm)	11	6–20
Mean height of the full thickness defect (mm)	4	1–12
Superficiel defect involving the alar rim	2	(22%)
Superficiel defect not involving the alar rim	8	(44%)
Distance from the alar rim (mm), mean	2	1–4
**Composite graft characteristics**		
Donor site		
Root of helix	17	(94%)
Posterior aspect of the auricle	1	(6%)
Closure of donor site		
Direct closure	14	(78%)
Local flap	4	(22%)
**Postoperative characteristics within 30 days**		
		
	5	(28%)
Partial loss of graft	3	(17%)
Complete loss of graft	1	(6%)
Re-operation	0	(0%)
Infection	0	(0%)
		
Donor site wound healing issues	0	(0%)
Number of out patient visits	3	1–5
**Follow up**		
Follow up, months, mean (range)	7	3–22
Revision surgery	2	(11%)
**Outcome**		
Same reconstructive choice in hindsight	18	(100%)
Overall outcome		
Poor	4	(22%)
Acceptable	5	(28%)
Good	9	(50%)

All cases were reviewed by first and last authors and categorized as whether a composite graft would have been chosen with our current knowledge, which was the case for all patients (100%). The outcomes were categorized as poor, acceptable, or good in four (22%), five (28%), and nine (50%) cases, respectively.

## Discussion

A composite graft for nasal reconstruction is a one-stage procedure which can be performed under local anesthesia as an out-patient procedure. Most cases in our series were secondary to cancer. As the surrounding tissue is left untouched, it leads to safe and easy cancer surveillance. If local recurrence is diagnosed; local tissue has been left uncompromised and is available for further reconstruction. In other parts of the world, the use of composite grafts has been described more often for reconstructions of nasal deformities due to burn injury and congenital defects [4,5] These grafts offer an alternative to full thickness skin grafts (FTSG), local and pedicled flaps for reconstruction of the lower third of the nose. With FTSG for deep defects at the lower third of the nose, a concave defect and retraction of the alar rim due to contraction of the graft usually occurs. While composite grafts are thicker than FTSGs, they do not cause concavity, and the grafts also provide a good colour and texture match to the skin of the nose. Additionally, they can also be employed at a later stage to remedy alar retraction, as illustrated in Figure 7. Compared to local flaps, such as bilobed, dorsal nasal or nasolabial flaps or pedicled flaps, an advantage of composite grafts is the donor site, away from the midface, avoiding potential blunting of the alar groove. The donor site, whether at the root of helix or posterior surface of the ear, usually heals well with minimal deformity and provides a good three-dimensional match for reconstruction of the nose. Composite grafts, furthermore, can provide skin for both the outer surface and inner lining for full thickness defects in a single-stage operation without recruiting local tissue. Whereas pedicle flaps used for both outer and inner lining, such as a paramedian forehead flaps, require multiple procedures, other local flaps such as a nasolabial flap using the abundant tissue superior to the defect as described by Hosaka et al. requires recruiting healthy adjacent tissue [6]. The location of the defects reconstructed in our series were the nasal alar and soft triangle. Composite grafts, however, are also useful for reconstruction of the columella [4,7]. The alar rim contains no cartilage, however, non-anatomical grafting of cartilage provides alar support to prevent alar notching and nostril collapse. Since local and pedicled flaps lack this structural support they may be too bulky and impair the airway which would then require revision surgery.

An alternative to excision of carcinomas of the nose and composite graft reconstruction is radiotherapy. Despite an often excellent functional and cosmetic outcome after radiation therapy, the treatment is time consuming, lengthy, and may cause long-term nasal crusting or dryness, postinflammatory hypo- or hyperpigmentation, atrophy, fibrotic tissue, alar retraction, and telangiectasia covering an area larger than the tumor site [8]. In a randomized controlled trial, comparing surgery and radiotherapy for BCC of the face, the recurrence rates after 4 years were 0.7% and 7.5% after surgery and radiotherapy, respectively [9]. Moreover, the cosmetic results after 4 years were better with surgery than with radiotherapy for the nose, however, not statistically significant [8]. A study by Schulte et al. evaluating soft x-ray therapy for epithelial malignancies, of which 489 were located on the nose, reported a total recurrence rate of 5% and observations during follow-up of hypopigmentation in 73%, telangiectases in 52%, erythema in 45% and hyperpigmentation in 23% [10]. Recurrence in the radiated field, must be managed by surgery, which is more complicated in irradiated tissue, with a higher risk of wound healing complications and hence, potentially a worse outcome.

Composite grafts are dependent on revascularization and bridging phenomenon over the cartilage for survival. It is therefore paramount for graft take that a vascularized wound bed is available for capillary ingrowth and that the graft is immobilized to avoid detrimental shearing forces causing graft failure. The former can be ensured by enlarging the surrounding superficial defect and to avoid excessive cautery to the wound bed. The maximum graft width in our series were just over two cm. As illustrated by the largest two defects in our series, a maximum distance of approx. 1 cm from a vascular source can be applied. This is in accordance with one of the most quoted measurements for the maximum size of a graft by Ruch stating that ‘no part of the graft should be much more than 1 cm from a free edge’ [2,11]. Others have, however, recommended to limit the size of composite grafts to one cm or less with no part of the graft more than 5 mm from a vascularized edge [12]. To enhance graft take, a combination of a hinge-flap elevated from the recipient defect margin can be used in conjunction with a composite graft [4]. Another way of improving the survival rate of the graft is delaying the reconstruction until the defect has healed partly by secondary intention providing granulation tissue [13]. This might, however, lead to distortion of the wound edges, and necessitate two procedures, and has not been used in our series. Others advocate adding a deepithelized dermal part of the graft to be inserted in a well-vascularized pocket to increase ingrowth of capillary network to supply the graft [5,14]. The disadvantage of this is the added bulkiness which might narrow the air flow through the nostril. Pilanc et al. attributed their success in reconstructing patients with nasal defects secondary to burns or facial clefts to using both a dermal turnover flap and the insertion of a deepithelialized part of the graft into a fold cranially to the defect [5]. Other attempts at improving the graft take, such as hyperbaric oxygen therapy, has been described [15]. When managing full-thickness defects, it is important to not only look at the maximum width of the full thickness defect but also the height. The largest full thickness defect in our series was in an 81-year-old woman, shown in Figure 3, developed partial necrosis of the central tissue furthest away from the vascularized wound bed. The result was rated as poor, however, the result was still a partial success, as the remaining composite graft did provide stable tissue for use of an exoprosthesis and for skin colored surgical tape that the patient considered to be more convenient to use. The patient declined the offer of a paramedian forehead flap. An equally large skin defect with a smaller full-thickness defect, in a 69-year-old man, survived with no tissue loss, as shown in Figure 8, illustrating the importance of well-vascularized tissue at the recipient site for revascularization of the graft. The defect had oblique margins, which provided this.

In smaller defects, where most of the skin graft overlies the cartilage, which acts as a barrier for imbibition, inosculation, and revascularization of the graft, the graft take, which is mostly at the margins of the defect, can therefore be more challenging than with larger grafts. In these cases, harvesting or leaving as little cartilage under the skin as possible, without losing its stabilizing properties, might enhance graft take as this would provide more surface area for revascularization. The thicker the graft, the higher the metabolic demand. As the subcutaneous tissue between the skin and cartilage can only be trimmed minimally not to affect the blood supply of the graft, choosing a donor site with the least subcutaneous tissue might improve graft take. In our series, most grafts were harvested from the root of helix where the subcutaneous tissue usually is only a few mm thick. If the defect is superficial and no inner lining is needed, the postauricular donor site is also an option. This site was used in one of our cases. A potential drawback of a composite graft is the long-term shrinkage or atrophy of the graft. In our series, the longest follow-up was 22 months. While no significant shrinkage or atrophy of the graft was noted during this period, our follow-up period does not allow for long-term assessment of the technique.

The literature on risk factors associated with composite grafts is scarce. In a study by Wang et al. assessing the outcome of reconstruction using flaps or grafts, former and current smokers, increased defect size, and free cartilage grafts were found to be associated with an increased rate of acute complications defined as any postsurgical infection, dehiscence, hematoma, uncontrolled bleeding, and tissue necrosis that required medical counselling or intervention [16]. In that study, only 13 patients were reconstructed with a free cartilage graft and details hereof were not available. We did not reconstruct current smokers with composite grafts; however, we did not distinguish between former or never smokers. Other potential risk factors associated with complications after reconstructive surgery are patient age, sex, comorbidities, such as diabetes and immunosuppression among the most important patient-related factors, and infection, poor recipient graft bed such as after radiotherapy, and mobilization, leading to shearing, are the most import wound-related factors. No infections were observed in our series. Nine (50%) patients were given prophylactic antibiotics, but none developed infections. Similarly, no hematomas occurred, despite one in three patients being on blood thinning agents, and only two patients had halted this before surgery. It has previously been stated, that composite grafts should be restricted to patients under 65 years of age with no systemic illnesses that would compromise graft revascularization [17]. In our series, the majority (12 (67%)) were ≥65 years old and only 6 (33%) had no comorbidities. Postoperative cooling has been described by some authors to slow down the biological demands of the graft until revascularization has occurred [4,14,18]. Only two patients were advised to use ice packs over the grafts (for 10 minutes every hour during the first 24 h when awake) hence no conclusion can be drawn based on this. The heterogeneity of the cases regarding both patients, surgeons, defects, and reconstructive parameters does not allow for assessing factors associated with success. All relevant parameters must be considered, and in our series, we did not see any trends justifying any age, comorbidity, or other patient-related variable cut-offs; however, defect selection is crucial when considering the use of a composite graft. In our experience, composite grafts are particularly suitable for superficial defects bordering or involving the alar rim and for reconstruction of full thickness defects, where the reconstruction was rated as good, when the maximum height was 4 mm, and the maximum width was 12 mm. For larger full thickness defects more complications must be anticipated. The failure rate is higher with composite grafts when compared to skin grafts or local flaps [16]. Epidermolysis was seen in some cases where reepithelialization led to good outcomes (not shown) whereas it in other cases (Figures 2–3) was seen leading to partial tissue loss and poorer outcomes. It is therefore important to be patient and take a ‘wait-and-see’ approach, of multiple weeks, before deeming failure of graft take as it can be slower, as illustrated by Figures 2–3, than what is seen with skin grafts. One patient (6%) experienced complete graft failure. This patient was an 87-year-old male, with a full thickness defect, where graft take was only possible from the margins of the defect (Figure 9). Patient-related factors, particularly his age and comorbidities (diabetes, ischemic heart disease and hypertension), might also have contributed to this failure. The surgical technique, however, could retrospectively have been modified enlarging the wound bed, potentially increasing the success rate. If surrounding healthy tissue had been excised, and larger wings had been used, the area for vascularization would have increased. This, however, would be at a cost of increasing the defect, which in case of graft failure would have left the patient worse off from a reconstructive point of view. The cartilage, furthermore, under the full-thickness graft, should also be kept as a minimum (max 3 mm in height) to increase skin graft take, particularly, for these smaller defects. Tissue loss, at the alar rim, is devastating and to avoid this complication a sufficient contact area for graft take is crucial.

None of our patients had received radiotherapy to the operating field. Using a composite graft in a radiated field may be associated with a higher risk of graft failure. It does, however, still have a place as the surrounding tissue is left untouched and if failure occurs, secondary procedures such as a forehead flap can be performed at no worse state. Others authors have, however, used composite grafts successfully following irradiation. [18]. The only absolute contraindication is therefore, the maximum size of the defect, if full-thickness, which should ideally be less than 4 mm in height, and 12 mm in width. If the graft is harvested from the anterior aspect of the ear the size of the graft harvested, without causing distortion of the ear, is a limiting factor. The use of composite grafts in irradiated tissue and in smokers is only relatively contraindicated.

Most of the grafts in this series were harvested from the root of the helix. Other parts of the ear, such as the antitragus or the posterior aspect of the ear, can also be used [7,19]. The root of helix was chosen due to factors related to the cartilage and skin. The cartilage of the crus of the helix has a natural curve imitating the alar rim. The cartilage is wide and a part of this can be harvested in the shape needed leaving enough support for the ear without noticeably affecting the appearance of the ear. The skin is typically abundant for these reconstructions as it can be harvested preauricular, as shown in Figure 1(B).

The grafts were made to fit the defect, which, was the right choice in most cases. In some cases, however, alar retraction was noted. This could be due to contracture of the graft, loss of tissue or insufficient stability of the cartilage. To address the former, the grafts could be designed slightly larger to accommodate the contracture. This might in some cases lead to bulkiness and thus a need for revision surgery. The instability of the cartilage caused by a mechanical pull or warping of the cartilage might also be alleviated by using a larger and more resistant cartilage graft (Figure 6(E)). Revision surgery was conducted in two patients due to partial tissue necrosis, and protrusion of the cartilage wings which caused discomfort to the patient (Figure 5). The first patient, who experienced tissue loss, was a healthy 63-year-old man, with no comorbidities and only a superficial defect. The graft seemed vascularized at the first dressing change seven days postoperative, and the patient was therefore only seen next after two months, at which point the distal part of the graft was non-vital. This patient underwent revision surgery with direct closure of the distal part of the defect. With composite grafts, which can be difficult to evaluate due to epidermolysis, a closer follow-up regime could be applied. The second patient, who underwent revision surgery, was displeased with the protruding cartilage (Figure 5). Minor alar rim retraction led to acceptable outcomes. It could possibly have been avoided by using wider cartilage limbs, which support the alar rim better, and by avoiding stitching up the graft and bolster dressing at the alar rim to prevent tissue necrosis surrounding the sutures and the sutures’ pull on the rim. Discoloration of the grafts was noted in one patient at the first follow up three months after surgery but at the last follow up twenty-two months after surgery the discoloration had subsided (Figure 6). This is in line with what can be expected, or even less than with a full-thickness skin graft and much less than what can be expected after radiation therapy [10]. All patients in our series were Caucasian. Hypo- and hyperpigmentation of the grafts were noted in a series of patients with darker skin tones, and they should be informed of this potential color mismatch [4].

With the increase of the older population, and the increase in non-melanoma skin cancer (NMSC) worldwide, particularly in the Caucasian populations, NMSC predilection for the nose and the decrease in available resources, composite grafts should be in the reconstructive surgeon’s toolbox as it offers an out-patient single-stage technique with excellent outcomes [20,21,22].

The technique can be performed by even less experienced surgeons, as illustrated by our series, where 2 (of 9) of the surgeons performed the reconstructions while in-training. We did, however, notice potential improvable outcomes in cases performed by surgeons with low number of cases, which was particularly evident for less experienced surgeons, which may be explained by the complexity of the procedure requiring more experienced surgeons, or simply a matter of a learning curve. Our eighteen cases were performed by nine different surgeons. This likely reflects the outcomes. With increase case numbers and experience the outcomes are anticipated to improve. All cases were reviewed by first and last authors and categorized as whether a composite graft would have been chosen, with our current knowledge, which was the case for all patients (100%). With our current knowledge, however, we would have made a few minor adjustments to our technique in some of the cases as described above.

The overall outcomes were categorized as poor, acceptable, or good in four (22%), five (28%), and nine (50%) cases, respectively. The four poor results were caused by partial or complete tissue loss, of which three patients declined revision surgery (Figures 3 and 9), are the main limitation of the use of composite grafts. These, to some, distracting outcomes are however acceptable in a population of patients where other reconstruction options are not feasible or in patients, who do not want to proceed with more complex reconstructions as illustrated by these three patients. The use of composite grafts would, however, allow for local or pedicled reconstructive options if the patients had opted for revision surgery. The alar retractions, which were partly responsible for the outcomes only rated as acceptable, might also have been encountered if the cases had been managed with radiotherapy or other surgical procedures (Figures 2 and 7). Half the patients had good outcomes with preserved or recreated alar rims, patent airway and contour of the alar (Figures 4–6, and 8). These successes can be attributed to the location and dimensions of the defect, the well-vascularized wound bed, factors related to wound healing, and the experience of the surgeons.

## Conclusion

In conclusion, many defects near or involving the alar rim, both superficial and full thickness, can be managed successfully with composite grafts, harvested from the auricle. In our experience, composite grafts are versatile and can be used for reconstruction of the lower third of the nose after oncological resection, and to correct collapse of the nostril and alar retraction. Thorough analysis of the location and the dimensions of the defect is crucial. Composite grafts are an excellent method of reconstruction to master for reconstructive surgeons; they are one-stage procedures with no additional midface scars, and the procedure can be carried out under local anaesthesia and is well tolerated by patients. This especially applies to defects at the alar rim or close to the alar rim. With through and through defects there are other good alternatives for reconstruction. These, however, often require multiple procedures, often under sedation or general anaesthesia. Many older patients as in our case series, need and/or prefer a simple immediate reconstruction, which can be performed in the outpatient clinic under local anaesthesia. Composite graft for alar reconstruction is thus a method that should be considered in selected cases. As with all reconstructions the choice of patient/defect is crucial when choosing the method of reconstruction.
